# Likelihood-free posterior estimation and uncertainty quantification for diffusion MRI models

**DOI:** 10.1162/imag_a_00088

**Published:** 2024-02-06

**Authors:** Hazhar Sufi Karimi, Arghya Pal, Lipeng Ning, Yogesh Rathi

**Affiliations:** Psychiatry Neuroimaging Laboratory (PNL), Brigham and Women’s Hospital, Harvard Medical School, Boston, MA, United States

**Keywords:** diffusion MRI, posterior estimation, uncertainty quantification, fiber reconstruction, parameter estimation

## Abstract

Diffusion magnetic resonance imaging (dMRI) allows to estimate brain tissue microstructure as well as the connectivity of the white matter (known as tractography). Accurate estimation of the model parameters (by solving the inverse problem) is thus very important to infer the underlying biophysical tissue properties and fiber orientations. Although there has been extensive research on this topic with a myriad of dMRI models, most models use standard nonlinear optimization techniques and only provide an estimate of the model parameters without any information (quantification) about uncertainty in their estimation. Further, the effect of this uncertainty on the estimation of the derived dMRI microstructural measures downstream (e.g., fractional anisotropy) is often unknown and is rarely estimated. To address this issue, we first design a new deep-learning algorithm to identify the number of crossing fibers in each voxel. Then, at each voxel, we propose a robust likelihood-free deep learning method to estimate not only the mean estimate of the parameters of a multi-fiber dMRI model (e.g., the biexponential model), but also its full posterior distribution. The posterior distribution is then used to estimate the uncertainty in the model parameters as well as the derived measures. We perform several synthetic and in-vivo quantitative experiments to demonstrate the robustness of our approach for different noise levels and out-of-distribution test samples. Besides, our approach is computationally fast and requires an order of magnitude less time than standard nonlinear fitting techniques. The proposed method demonstrates much lower error (compared to existing methods) in estimating several metrics, including number of fibers in a voxel, fiber orientation, and tensor eigenvalues. The proposed methodology is quite general and can be used for the estimation of the parameters from any other dMRI model.

## 1 Introduction

Unlike histological techniques, diffusion magnetic resonance imaging (dMRI) enables researchers to study the microstructural properties of brain tissues in a noninvasive fashion. Furthermore, the brain’s structural connectivity can be efficiently mapped via dMRI-based tractography strategies (Malcolm et al., 2010; Tournier et al., 2019; F. Zhang et al., 2022). The diffusion signal can be described as a function of its model parameters (e.g., number of fibers, orientation, and diffusion coefficient). Accurate estimation of these parameters is paramount for any neuroimaging study, especially when it involves calculating statistics of dMRI-derived measures for a fiber bundle or region-of-interest (ROI) in the brain.

### 1.1 Literature review

In recent decades, there have been several models and tools used to estimate the fiber orientation and the underlying microstructural measures of brain tissue. Generally, the proposed models are categorized into two groups: 1) signal representation and 2) biophysical models. In the signal representation models, diffusion tensor imaging (DTI) is the simplest and the most popular one, where diffusion is modeled assuming a single fiber orientation with a Gaussian distribution (Basser et al., 1994). While it is an effective approach in some cases, DTI fails to adequately describe the underlying structure if a voxel contains multiple fiber populations or complex architectures (D. Alexander et al., 2002). Therefore, subsequent works (e.g., Dell’Acqua et al., 2010; Kreher et al., 2005; Peled et al., 2006; Rathi et al., 2009; Sid et al., 2017; Tuch et al., 2002) focused on addressing these challenges by introducing more complex diffusion models. As an expansion of DTI, Diffusion Kurtosis Imaging (DKI) quantifies the non-Gaussian diffusion in biological tissues (Jensen et al., 2005) that is compatible with crossing fibers (Henriques et al., 2015), and results in a better characterization of tissue microstructural heterogeneity (Jensen & Helpern, 2010). Instead of directly estimating the number/direction of the fibers, an orientation distribution function (ODF) is estimated that provides the probability of diffusion in various directions (Tuch, 2004). The most pupolar among these methods uses the spherical harmonic basis (Descoteaux et al., 2007; Hess et al., 2006) to represent the ODF. Performing spherical deconvolution and modeling the signal response from a single-fiber allows one to compute the fiber-ODF (or fODF) (Aganj et al., 2010; Dell’Acqua & Tournier, 2019; Jian & Vemuri, 2007; Kaden et al., 2007). Finally, a 3-dimensional ensemble average diffusion propagator was proposed (Merlet & Deriche, 2013; Ning, Westin, et al., 2015; Özarslan et al., 2013) to compute the 3-dimensional probability distribution of the displacement of water molecules.

Another category of diffusion models aim to quantify the biophysical structure of the tissue in the brain. In H. Zhang et al. (2012), the authors introduce a new diffusion model known as neurite orientation dispersion and density imaging (NODDI) that can portray the geometrical angular variation of the fiber populations via a fiber orientation dispersion index. Other recent models include the standard model of diffusion and models that use specialized b-tensor encoding sequences (Coelho et al., 2022; Jelescu et al., 2016).

A comprehensive review of different diffusion models, analytical comparisons of the techniques, and existing challenges are provided in D.C. Alexander (2005), Assemlal et al. (2011), Descoteaux et al. (2008), Jelescu and Budde (2017), Novikov et al. (2019), and Panagiotaki et al. (2012). Histological validation and evaluation of various dMRI techniques can be found in Novikov et al. (2018) and Schilling et al. (2018). The sensitivity of diffusion MRI to microstructural properties and experimental factors is reviewed in Afzali et al. (2021).

Most existing methods use nonlinear least squares (or similar) optimization techniques to estimate the model parameters. However, given that the dMRI signal has a very low signal-to-noise ratio (SNR), the estimation of these parameters can vary significantly depending on the initialization of the optimization algorithm or the minimization method used (Jelescu et al., 2016; Jones & Basser, 2004; Koay & Basser, 2006). Some recent works have tried to address this issue using machine-learning techniques (Gibbons et al., 2019; D. Karimi et al., 2021; Tian et al., 2020), however, none of these methods address the problem of uncertainty quantification. Further, these methods do not consider the problem of crossing fibers in the estimation of the model parameters, which dramatically increases the complexity of the inverse problem.

Given the low SNR, characterization of the uncertainty in estimation becomes paramount for robust post-processing and analysis. For example, the mean FA can be highly biased if several voxel-wise estimates of eigenvalues are inaccurate, thereby reducing statistical power and increasing the possibility of false positive results when performing group analysis. To cope with the Rician noise in dMRI (Gudbjartsson & Patz, 1995), a method using feature fusion and attention network (FFA-DMRI) was proposed to account for high noise in the measured dMRI data (Hong et al., 2020). Some methods have been proposed to estimate the cone of uncertainty (Koay et al., 2008; Malcolm et al., 2010; Reddy & Rathi, 2016) using error propagation methods or within an unscented Kalman filter based tractography method. However, they only estimate the covariance between the relevant parameters and do not provide access to the full posterior distribution, that is, the joint distribution between the parameters of the model (which could be high dimensional). Uncertainty calculation is particularly relevant for dMRI tractography, which is associated with the problem of tracing false positive tracts (Maier-Hein et al., 2017).

In dMRI, the forward model is well-defined; however, the inverse problem is plagued with ambiguity as several likely solutions might exist (Ardizzone et al., 2018; Jelescu et al., 2016; Novikov et al., 2018; Scherrer & Warfield, 2010). Knowledge of the full posterior distribution of the model parameters can help resolve this ambiguity and enable a better understanding of the model uncertainties. Quantification of this uncertainty and its use in calculating robust statistics in neuroimaging studies will help reduce false positive results and could be of great use when performing tractography. Machine learning has become an effective approach in calculating uncertainty and its application. For example, Reinhold et al. (2020) designed a Bayesian deep learning method that learns to estimate voxel-wise uncertainty for pathological purposes. In a recent work, Jallais et al. (2021) employ a normalized flow technique to solve the inverse problem of grey matter modeling. In Tanno et al. (2021), the authors presents a strategy for uncertainty modeling in deep learning via two different signal representations of dMRI. To the best of our knowledge, characterizing the full posterior distribution of the diffusion model parameters has not been investigated yet. Therefore, this paper aims to bridge this gap.

### 1.2 Contributions

We propose several novel contributions in this work, namely: (i) a deep learning method to accurately estimate the number of principal fiber orientations per voxel, (ii) a likelihood-free inference method to estimate the full posterior distribution of the underlying model parameters (despite the large dimensionality of problem), and (iii) a method to estimate the uncertainty in the model parameters as well as the derived measures, which has not been done before beyond the DTI model.

To fully characterize the uncertainty associated with the estimated parameters of a dMRI model, estimation of the full posterior distribution (conditioned on the observed measurement) is required. However, the number of parameters to estimate depends on the number of fibers within each voxel (assuming a discrete number of fiber orientations per voxel). Thus, one has to first solve the model selection problem before addressing the problem of estimating the full posterior distribution of the model parameters. To do so, we develop a voxel-wise classifier mechanism that categorizes the dMRI signal based on the possible number of principal fiber orientations. This classifier takes the ODF of each voxel and passes it to a convolutional neural network (CNN), which determines the number of crossing fibers at that voxel. Next, we employ a likelihood-free inference approach, which allows for estimating the full posterior distribution of the underlying model parameters at that voxel using a neural network (NN) setup. Note that, the number of model parameters to estimate can be large and increases linearly with the number of fibers present in each voxel. Training can be easily done in this method using simulated samples that are obtained via the forward model. Third, we use an unscented transform to derive analytical expressions for uncertainty (quantified using the variance) in some of the derived dMRI measures (e.g., FA, RTOP, MSD). The proposed approach was evaluated using both synthetic data and in-vivo human brain datasets. Thus, a key novel aspect of our work includes development of a fast algorithm to estimate the full posterior distribution of the model parameters and subsequently of the derived measures (which are nonlinear functions of the model parameters). The proposed work will be very useful in calculating robust statistics in dMRI studies. While we demonstrate the power of this method using a biexponential multi-fiber model, the methodology is quite general and can be used for any dMRI model (H.S. Karimi et al., 2023).

### 1.3 Paper organization

This paper is arranged in the following manner: Section 2 is dedicated to discuss our method where subsection 2.1 describes the diffusion model and the transformation of the ODF into 2D space for efficient use with an NN formulation. In subsection 2.2, we explain our approach and discuss how we configure the NN for classification and likelihood-free posterior estimation. Section 3 evaluates performance and demonstrates the results on both synthetic and in-vivo data. Finally, section 4 provides a summary of this paper.

## 2 Methods

### 2.1 Preliminaries and problem statement

#### 2.1.1 Diffusion model

To characterize the posterior distribution, we need access to an accurate propagator that models the fibers’ desired parameters (e.g., orientation). Several models have been proposed in the literature (Panagiotaki et al., 2012) with varied number of model parameters. In general, the signal at a voxel can be defined as $S\left(b,u\right):\mathbb{R}\times S^{2}\to {\mathbb{R}}^{+}$ that measures diffusion along a set of distinct gradient directions u. Here, b is an acquisition-specific constant (known as b-value) and normally varies between 1000 and $3000\;s/mm^{2}$. Based on the diffusivity, the diffusion signal decays as a function of b-values where the signal decay can be approximately modeled by a mono-exponential (Gaussian) at low b-values. However, such a model cannot accurately predict signals at higher b-values, as the signal decay demonstrates a bi-exponential behavior (Assaf et al., 2004; Clark & Le Bihan, 2000; Maier et al., 2004; Mulkern et al., 1999; Novikov et al., 2018). Note that several other models, such as NODDI (H. Zhang et al., 2012) and DKI (Jensen et al., 2005), impose strong conditions within their models to allow for proper estimation of parameters. However, this limits their ability to generalize to all possible scenarios. For example, different values of intra-cellular and extra-cellular terms or the interchangeability of the tensors may result in similar signals, which are hard to interpret. On the other hand, estimation of the parameters of a more flexible biexponential model is considered challenging due to the possibility of a non-unique solution set. Thus, we choose this model to demonstrate the power of the proposed technique in characterizing the full posterior and how it can be used for choosing the right solution from different possibilities.

It should be noted that the dMRI signal contains multiple diffusing compartments (e.g., fast/slow diffusion fractions). Furthermore, most voxels contain a mixture of multiple fiber orientations crossing at different angles. Since our goal is estimating the uncertainty in the microstructural parameters in a fiber-specific manner, we assume a discrete number of fiber population in a voxel. Therefore, we use the following weighted Gaussian formulation proposed in Rathi et al. (2013) to model the dMRI signal:

$$S(b,u)=\frac{1}{4}\sum_{i}^{n}\omega_{i}e^{-bu^{T}D_{i}u}+(1-\omega_{i})e^{-bu^{T}{\bar{D}}_{i}u}$$ (1)

where n indicates the number of fibers, and $\omega_{i}$ is the coefficient of the fast diffusing component. Furthermore, $D_{i}$ and ${\bar{D}}_{i}$ denote the fast and slow diffusion tensors sharing the same eigenvectors but different eigenvalues with $D_{i}=\lambda_{1i}\;m_{i}m_{i}^{T}+\lambda_{2i}\left(p_{i}p_{i}^{T}+v_{i}v_{i}^{T}\right)$ and ${\bar{D}}_{i}=\lambda_{3i}\;m_{i}m_{i}^{T}+$$\lambda_{4i}\left(p_{i}p_{i}^{T}+v_{i}v_{i}^{T}\right)$. In this formulation, $m_{i},p_{i},v_{i}\in S^{2}$ are three orthonormal bases of the $i^{th}$ tensor where $m_{i}$ represents the principal diffusion direction. Here, we assume that the tensors have cylindrical symmetry, that is, the tensors have a single dominant eigenvalue with the remaining two eigenvalues being equal. Accordingly, the model consists of the following parameters: $x_{i}=\left[m_{i},\;\lambda_{1i},\;\lambda_{2i},\;\;\lambda_{3i},\;\;\lambda_{4i}\right]$. Furthermore, since $m_{i}$ is a unit vector, it can be represented by two angles in spherical coordinates [θ,ϕ]. Due to antipodal symmetry of the ODF, the range of θ,ϕ can be restricted to the right hemisphere, that is, θ,ϕ∈[0,π]. In summary, the model parameters can be reduced to $x_{i}=\left[\theta_{i},\phi_{i},\lambda_{1i},\lambda_{2i},\lambda_{3i},\lambda_{4i}\right]$∀i∈{1,...,n}. Therefore, the complete parameter set for the multi-fiber case is $\alpha =\left[x_{1},x_{2},...,x_{n}\right]$, where n is the number of crossing fibers and should be determined for each voxel.

In this work, we use HCP style acquisition parameters with 90 gradient directions at b-values of {1000, 2000,3000}. The same set of parameters were used in our simulation experiments. Note that since our main focus in this paper is dedicated to white matter, our model does not include water exchange, which is an essential factor in modeling gray matter (Jelescu et al., 2022; Olesen et al., 2022).

#### 2.1.2 ODF representation

Typically, dMRI studies (across centers) do not acquire the same number (or set) of gradient directions as it is dependent on the scan time budget and analysis methods used. Therefore, using a fixed gradient direction set will restrict the use of a trained NN to that particular set and hence cannot be generalized to the typical scenarios of neuroimaging studies. To address this issue and to ensure generalization of our framework for any number of gradient directions, we use the diffusion oriented distribution function (ODF) representation of the signal S. In this paper, we compute the ODF using spherical harmonics (SH) (Hess et al., 2006). Spherical harmonic transform can be interpreted as a generalized form of the Fourier expansion to the domain of the sphere (Groemer, 1996). Calculating accurate signal representation demands that the harmonic series order L be sufficiently large. On the other hand, the order L is limited by the number of measurements. For example, using spherical harmonics up to order L=8 requires at least 45 distinct diffusion measurements. However, we note that the connection between the accuracy of a SH fit and its relation to the number of measurements available for estimation is a research topic on its own and has been explored in the following works (Consagra et al., 2023; Descoteaux et al., 2007; Deriche et al., 2009). In this work, we assume that an appropriate SH fitting algorithm is available to estimate the ODF at each voxel. Notably, newer methods of ODF or fODF estimation can be easily incorporated into our work.

Once the ODF is computed, a hill-climbing algorithm is typically used to calculate the ODF peaks, which represent the principal diffusion directions of the underlying fibers. However, the number of fibers and the orientations are often inaccurately estimated due to several factors such as measurement noise, close crossing angles, and different levels of diffusivity (Ning, Laun, et al., 2015). Furthermore, estimation only based on raw ODFs is extremely unreliable in the case of fibers crossing at low to moderate angles. Therefore, it is very critical to develop a robust computational technique that can efficiently classify the voxels based on the number of fibers they contain and estimate the fiber orientations.

**Fig. 1.**
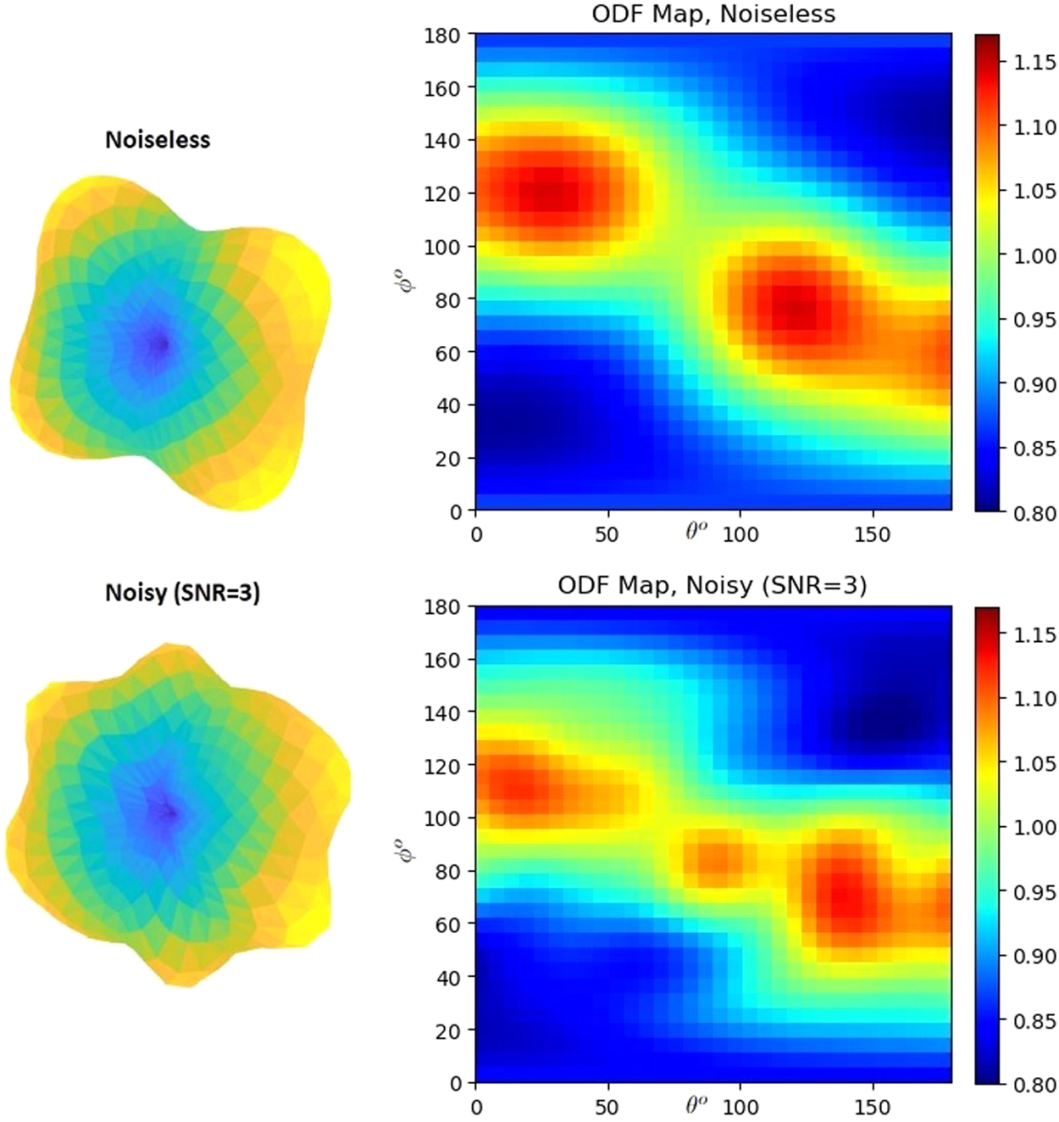
ODF representation (Right: 2D spherical coordinate ODF map, Left: three-dimensional ODF) of a voxel containing two fiber crossing, when the underlying signal is noiseless (top), and noisy (bottom) with SNR = 3.

In diffusion MRI, the measurement noise associated with the diffusion signal is a potential challenge that can impair many of the existing reconstruction methods. Noise distribution depends on many factors, including number of receiver coils, spatial resolution, and gradient strength among others (Gudbjartsson & Patz, 1995; St-Jean et al., 2020). Throughout this paper, SH coefficients corresponding to synthetic data are computed using S+%, where % is zero-mean Gaussian noise with a certain standard deviation. Figure 1 shows a synthetic example of an ODF without and with noise (SNR = 3) which notably distorts the original ODF (top left) causing several false peaks (bottom left) and significant deviation from the ground truth. This demonstrates the need for a robust technique for accurate fiber orientation as well as parameter estimation from highly noisy dMRI data (Jones & Basser, 2004).

By converting the 3D representation of the ODF in spherical coordinates, we can create a 2D map of the ODF. Right pictures in Figure 1 show the transformed ODF map in 2D in the spherical θ,ϕ coordinate system of the same underlying ODF on the left side of Figure 1. For the rest of this paper, we will use this 2-dimensional ODF map as input to the designed DNNs. This strategy has two main advantages: 1) The neural networks dealing with these 2-D maps can extract more informative features and 2) Our neural network formulation will be relatively independent of the number of gradient directions.

### 2.2 Approach

In addition to the parameter estimation, quantifying the uncertainty of the estimated parameters can give us valuable insights into the reliability of the estimation in different regions of the brain. To do so, we aim to quantify the full posterior distribution of the parameters, that is, the probability of the parameters α given the observed measurements y, that is, p(α  |  y). Furthermore, since the configuration of the parameter vector and the underlying diffusion model changes based on the number of fibers in a voxel, we dedicate subsection 2.2.2 on finding the number of possible fibers in a voxel. In subsection 2.2.4, we introduce some well-known measures that can be derived from the biexponential model.

Estimating the parameters of a model is typically done via optimizing a loss function (e.g., an $L_{2}$ loss function) to estimate the maximum a-posteriori estimate. Another work such as Koay et al. (2008) derived an analytical cone of uncertainty while estimating the posterior of the DTI model parameters. On the other hand, the proposed work estimates the full posterior distribution of the model parameters (which could be multimodal and of high dimensionality) which implies an analytical and closed form joint distribution of all the model parameters. This extends the uncertainity quantification to arbitrary forward models of dMRI, without the need for complicated derivations of the cone of uncertainty as in Koay et al. (2008).

#### 2.2.1 Posterior estimation

Suppose we can correctly characterize the posterior probability of the parameters. In that case, we will be able to observe the behavior of the parameters based on a given set of measurements for a particular voxel. To find an analytical form of the posterior distribution, we need to calculate the likelihood p(y | α) implied by the Bayes theorem (i.e., $p(\alpha \;|\;y)=\frac{p(y\;|\;\alpha )p\left(\alpha \right)}{p\left(y\right)}$). However, computing the likelihood is not feasible since it requires calculating closed form solution of many intractable integrals (Papamakarios et al., 2019). To tackle this issue, Approximate Bayesian Computation (ABC) techniques were proposed as likelihood-free statistical inference approaches (Sisson et al., 2018). However, these approaches are not typically applicable for high dimensional cases. In addition, the required summary statistics and distance functions are customized based on ad-hoc options (Greenberg et al., 2019). On the other hand, the methods based on likelihood-free inference (Greenberg et al., 2019) amortize the posterior without any additional inference steps. These approaches assume that the forward model that maps the parameters α to the output data y is known, and that a sufficiently large training dataset can be simulated. Then, the posterior is directly extracted by a density-estimation NN trained using the simulated dataset.

Likelihood-free inference approaches approximate the true posterior density using a family of densities $q_{\psi }$, where ψ denotes distribution parameters. Let $\tilde{p}(\alpha \;\;\;\;|y)$ indicate the estimated posterior, then,

$$\tilde{p}(\alpha \;|\;y)=q_{\psi }\approx p(\alpha \;|\;y).$$ (2)

An NN configuration must be designed to estimate the density parameters ψ correctly. In the literature, several families $q_{\psi }$ have been proposed, including Mixture density networks (MDN) (Bishop, 1994), Masked Autoregressive Flow (MAF) (Papamakarios et al., 2017), Masked Autoencoder for Distribution Estimation (MADE) (Germain et al., 2015), and Neural Spline Flow (NSF) (Durkan et al., 2019). For our diffusion MRI model, we use the MDN as a representative of the true posterior. The MDN can be represented as a linear combination of m kernel functions $G_{i}(\alpha \;|\;y)$ as follows,

$$q_{\Psi }=\sum_{i=1}^{m}w_{i}(y)G_{i}(\alpha |y),$$ (3)

where $w_{i}(y)$ are known as mixing coefficients and control the contribution of the corresponding component $G_{i}(\alpha \;|\;y)$. Although numerous choices are available for the kernel functions, it has been shown that the kernels of Gaussian form result in a better approximation of the posterior as it can approximate any density function if the Gaussian parameters are correctly estimated (McLachlan & Basford, 1988). Therefore, we use the following form for the kernel function:

$$G_{i}\left(\alpha \;\text{|}\;y\right)=\frac{1}{{\left(2\pi \right)}^{\frac{C}{2}}|\Sigma_{i}\left(y\right){|}^{\frac{1}{2}}}\times$$

$$exp\left(-\frac{1}{2}\left(\alpha -\mu_{i}\left(y\right)\right)\Sigma_{i}^{-1}\left(y\right){\left(\alpha -\mu_{i}\left(y\right)\right)}^{T}\right)$$

where $\mu_{i}$ is the vector containing the center of $i^{th}$ kernel (Gaussian component). Here, $\Sigma_{i}$ represents the full covariance matrix of $i^{th}$ component. In Bishop (1994), a common variance $\sigma_{i}$ is introduced to describe the distribution of a component. However, we consider a more general case where the elements of a component can adopt different distributions while the covariance between the elements can be extracted from $\Sigma_{i}$. It should be noted that the number of kernels must be adequate to fully characterize the actual posterior density. After determining m, we employ a deep Neural Network (DNN) structure to approximate the parameters of the proposed density. Based on the MDN model described by (3) and (4), the required parameters are: $\psi =\left\{w_{i},\mu_{i},\Sigma_{i}\right\}\;\forall i\in \left\{1,...,m\right\}$.

In the training stage, we first simulate a dataset containing N pairs of $\left(\alpha_{j},y_{j}\right)$. Next, we train the DNN that approximates the posterior using the following loss function proposed in Greenberg et al. (2019):

$$L=-\sum_{j=1}^{N}\log\{{\tilde{q}}_{\psi }(\alpha_{j})\}$$ (4)

where, ${\tilde{q}}_{\psi }(\alpha_{j})=q_{\psi }(\alpha_{j})\frac{\tilde{p}(\alpha )}{p(\alpha )}\frac{1}{Z(\psi )}$. Here, p(α) and $\tilde{p}\left(\alpha \right)$ refer to the prior probability of the parameters and its estimation. In (4), Z(ψ) is a normalization constant and it equals to $Z\left(\psi \right)=\int_{\alpha }\;q_{\psi }(\alpha_{j})\frac{\tilde{p}\left(\alpha \right)}{p\left(\alpha \right)}$.

#### 2.2.2 Voxel classification

**Fig. 2.**
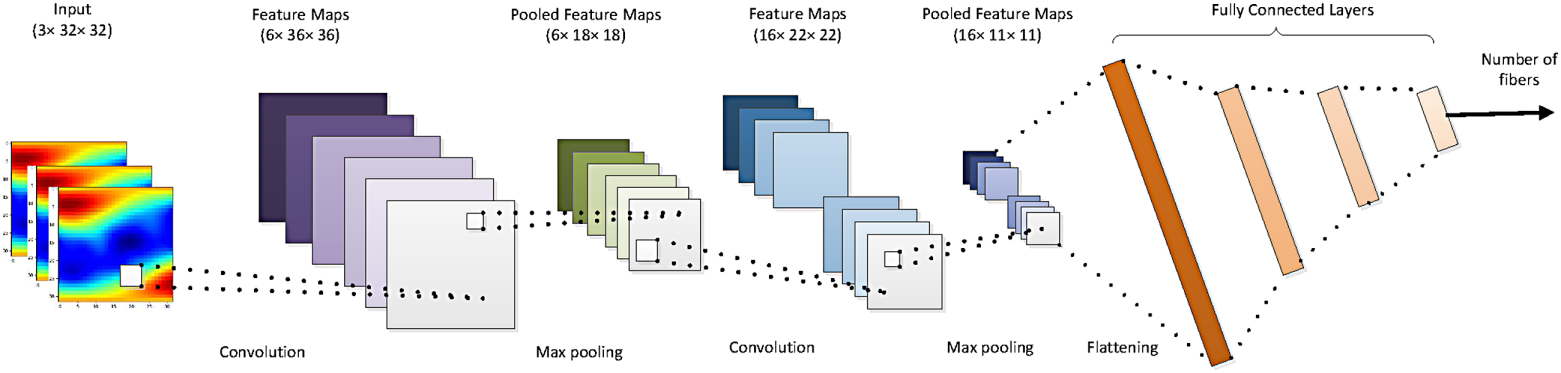
Classifier network architecture for estimating the number of fibers in each voxel. It includes two CNN downsampling layers followed by four fully connected layers.

The underlying diffusion model (1) (that we employed for simulating the training dataset) directly depends on the number of fibers in a given voxel (although methods exist to represent them in a continuous fashion). Although determining the number of fibers seems like a straightforward task, it has received significant attention from the research community as it fails in many cases due to measurement noise, close crossing angles, and different levels of diffusivity along different fiber bundles (Ning, Laun, et al., 2015). To overcome this issue, we design a DNN (Albawi et al., 2017) that classifies the voxels into three classes with 1, 2, or 3 fibers (from the 2D ODF map images). To this end, we implement a DNN, as shown in Figure 2, composed of a convolutional neural network (CNN) followed by four fully connected layers. In this paper, we assume a voxel has at most three fiber crossings, a reasonable assumption as more than three fiber crossings have not been validated from histology work. The input are 32×32×3 ODF maps, with the third dimension corresponding to b-values of $1000,\;\;2000,\;\;3000\;s/mm^{2}$. In addition to the fact that our experimental data has these three b-values (Scherrer & Warfield, 2010) show that multiple b-values are necessary for proper estimation of multi-fiber models. Next, a two-dimensional convolution layer extracts six feature maps using a convolution kernel size of 5×5. In order to not miss the information embedded near the image borders, we add a padding of size 4. Then, a max-pooling layer downsamples the resulting feature maps with kernel = 2 and stride = 2. A similar structure constitutes the two subsequent layers. The output of the last max-pooling layer is flattened and fed into a linear layer. The number of hidden features in the consecutive linear layers are set to be 1000, 500, and 84, respectively. The final section of the classifier structure consists of four fully connected layers that generate the labels, indicating the number of fibers. For the training stage, we employ a stochastic gradient descent optimization to minimize a cross-entropy loss function. Here, we choose the momentum to be 0.95, and the learning rate of $10^{-4}$.

#### 2.2.3 Configuration

We assume the parameters α follow a uniform prior distribution within a certain plausible range. That is, we only know the range of the parameters, but we do not assign any higher probability to a particular region between the minimum and maximum points. As described in section 2.1, direction of fibers can be fully expressed by θ,ϕ∈[0,π]. In addition, the fast eigenvalues $\left\{\lambda_{1},\lambda_{2}\right\}$ fall in the range of $\left[1,3\right]\mu m^{2}\;/\;\;ms$ and the slow eigenvalues $\left\{\lambda_{3},\lambda_{4}\right\}$ take a value in the range of $\left[0.1,0.6\right]\mu m^{2}\;/\;ms$. We also assume that the fast and slow components have 0.7 and 0.3 as their weights (Rathi et al., 2013).

The number of Gaussian components in the MDN model plays a significant role in acquiring a meticulous posterior estimation. Ideally, the existence of at least n! components is necessary for describing the desired posterior in a voxel with n fibers. To clarify this fact, consider a voxel with two fibers crossing each other. Let the parameter vector be $T_{1}=[\theta_{1},\phi_{1},\lambda_{11},\lambda_{21},\lambda_{31},\lambda_{41},\theta_{2},\phi_{2},\lambda_{12},\lambda_{22},$$\lambda_{32},\lambda_{42}]$ and $s_{1}$ is the expected noiseless signal. Now, if we swap the parameters corresponding to each fiber, (i.e., $T_{2}=[\theta_{2},\phi_{2},\lambda_{12},\lambda_{22},\lambda_{32},\lambda_{42},\theta_{1},\phi_{1},\lambda_{11},\lambda_{21},\lambda_{31},\lambda_{41}]$), the expected signal will still be $s_{1}$. This is due to the interchangeability property of the model (1). Accordingly, if the underlying voxel contains two fibers, one can expect two scenarios for the output vector. Similarly, six scenarios are possible for the case of three fiber crossings. In addition, extra numbers of components will be helpful to cope with the effect of measurement noise and outliers. As a rule of thumb, we consider five components for the one and two fiber voxels, while we consider 10 components for the voxels with three fiber crossings. Since we use a discrete representation for the number of fibers, the posterior distribution is conditioned on the number of fibers per voxel, which we estimate using our classification algorithm.

Similar to the classifier network, the DNN dedicated to posterior estimation consists of a convolutional network and a chain of seven fully connected linear layers. Determining the structure of the fully connected layers is very important and it influences the posterior estimator’s performance. Our brute force experiments to determine the best network architecture showed that a small number of layers (less than five layers) results in poor performance, while a large numbers of layers makes the network more complex and leads to over-fitting. We found that seven layers produced the most accurate results. Another factor affecting the performance is the number of hidden features in each layer. Here, we set the hidden features to be 1000 (after experimenting with different numbers). To avoid overfitting, we also embed dropout layers into the DNN.

We use 2 million simulated samples for training, and 10% of this data is used for validation. The training epochs continue until the validation loss decreases, and we stop the training when the validation loss starts to increase. In order to avoid being stuck in a local minimum, we consider 30 more epochs after the last increments. If we reach a new global minimum, we continue the training; otherwise, we select the computed parameters in the minimum validation loss stage. Here, we set the training batch size to be 100. Setting the learning rate to $5\times 10^{-5}$ was found to be most optimal. The entire code was implemented in python. Specifically for the training of the posterior, we used the python package provided by Tejero-Cantero et al. (2020).

#### 2.2.4 dMRI measures

Several measures have been proposed in the literature to better understand the underlying tissue microstructure (Assaf et al., 2004; Jensen et al., 2005; Ning, Westin, et al., 2015; Ning et al., 2017). For the case of multi-fiber models (like the model used in this work), these measures can be calculated for each fiber separately. Let $\lambda_{i}=[\lambda_{1i},\lambda_{2i},\lambda_{3i},\lambda_{4i}]$ denote the vector of eigenvalues for fiber i, and $\lambda =[\lambda_{1},...,\lambda_{n}]$ denote the vector of eigenvalues for a multi-fiber voxel. To evaluate the diffusivity of tensors, we compute Fractional anisotropy (FA) as defined in Basser and Pierpaoli (2011). Specifically, the FA of fast diffusing component corresponding to the $i^{th}$ fiber can be calculated using

$$FA\left(\lambda_{i}\right)=\sqrt{\frac{{\left(\lambda_{1i}-\lambda_{2i}\right)}^{2}}{\lambda_{1i}^{2}+2\lambda_{2i}^{2}}}.$$ (5)

Similar to FA, Tuch (2004) defines Generalized Fractional Anisotropy (GFA), a scalar measure for determining the level of isotropy in a multi-fiber voxel. GFA can be numerically computed as follows:

$$GFA\left(S\right)=\frac{std\left(S\right)}{rms\left(S\right)},$$ (6)

where std(S) and rms(S) are the underlying signal’s standard deviation and root mean square.

Another metric is Mean-Squared-Displacement (MSD), which expresses the average squared displacement during the diffusion experiment (Ning, Westin, et al., 2015). For the dMRI model (1), the MSD can be analytically computed using:

$$MSD(\lambda )=\frac{1}{n\pi^{2}}\sum_{i}^{n}w_{i}(2\lambda_{2i}\lambda_{1i})+(1-w_{i})(2\lambda_{4i}\lambda_{3i}).$$ (7)

Return to origin probability (RTOP) (Assaf et al., 2004; Baxi et al., 2020; Özarslan et al., 2013) serves as another measure that gives us an insight into restricted diffusion in different regions of the brain. RTOP can vary depending on changes in intracellular and intra-axonal or restricted spaces of white and gray matter tissue (Ning, Westin, et al., 2015). For our model (1), the RTOP can be computed analytically using:

$$RTOP(\lambda )=\frac{2}{n}\pi^{\frac{3}{2}}\sum_{i}^{n}\frac{w_{i}}{\lambda_{2i}\sqrt{\lambda_{1i}}}+\frac{1-w_{i}}{\lambda_{4i}+\sqrt{\lambda_{3i}}}.$$ (8)

An important contribution of this work is that we not only estimate the “mean” but also the uncertainty measured using the standard deviation (or variance) of these measures. Although the output of the DNN provides the posterior distribution for the model parameters, we approximate the posterior distribution of the derived measures up to second-order statistics (i.e., variance) using the unscented transformation as described in the Appendix.

## 3 Results

This section presents a comprehensive evaluation of our proposed method from different perspectives based on in-vivo and synthetic data. We examine our techniques using different synthetic datasets to validate our work and compare it with the ground truth data. To this end, we first generate the simulation data along 90 gradient directions at three different b-values $\left\{1000,2000,3000\right\}\;\;s\;/\;mm^{2}$. We added Gaussian noise to the data in all our simulation experiments with a standard deviation of noise being 0.04 (which provides an average SNR of 3 in the white matter). Note that, MRI noise follows a Rician distribution, but for SNR>3 the noise can be approximated with a Gaussian distribution as shown in Gudbjartsson and Patz (1995). For the simulations, we used the acquisition parameters from the human connectome project (HCP) dataset. At the training stage, we use a machine equipped with two GPU cores (CUDA = 11.5 with 22 GB total memory), which results in a big improvement in reducing the training time. With this machine configuration, the training times are 3.8, 11.3, 7.6, and 4.1 hours for one, two, three fiber crossing samples and the classifier, respectively.

### 3.1 The classifier performance

Table 1 summarizes the classifier’s performance, where our method could estimate the number of fiber crossings at any given voxel with a low percentage error even when the measured signal is very noisy and the crossing angles are small. The test dataset (20,000 test samples) was generated to mimic realistic scenarios seen in the human brain. It should be noted that the test dataset follows uniform distributions for all the parameters, and they have not biased toward any particular configuration of the parameter distribution. We used FA>0.2 for the case of two or three fibers crossings in the white matter. For the case of one fiber, the tensor was allowed to be completely isotropic, which means FA∈[0,1]. The crossing angle for the two fiber samples was chosen randomly between 10 and 90 degrees (note that very small crossing angle between the fibers is allowed). For the case of three fiber crossings, the minimum angle between the fibers was set to 45 degrees (based on realistic values reported in the literature). For comparison with existing techniques, we calculate the number of peaks using the state-of-the-art method called Constrained Spherical Deconvolution (CSD) (Tournier et al., 2007) as implemented in the DIPY package (Garyfallidis et al., 2014). As seen in Table 1, our proposed classifier dramatically outperforms the CSD technique in terms of identification of the true number of fibers. The much higher percentage of error in the CSD method can be attributed to the high noise levels (SNR = 3) as well as small crossing angles used in our simulations. We also note that we classified a voxel as “mis-classified” if the exact number of fibers were not estimated by the method. Thus, spurious peaks (even if they are small) provided by CSD contributed to the high mis-classification percentage for this method.

**Table 1.** Performance of the classifier for biexponential model.

Ground truth	1 Fiber	2 Fibers	3 Fibers
Misclassification (SNR = 3) (Our Approach)	2.7%	2.3%	4.7%
Misclassification (SNR = 3) (CSD)	45.8%	68.3%	91.6%
Misclassification (noiseless) (Our Approach)	0.18%	0.11%	0.34%
Misclassification (noiseless) (CSD)	26.6%	24.2%	50.2%

To demonstrate generalizability of the classifier to data from other dMRI models, we also generated data from the standard model of diffusion (Jelescu et al., 2016) and tested the classifier performance without any retraining. Note that the classifier was trained using the biexponential model. Table 2 shows the classifier performance using this dataset. Clearly, the performance is quite similar to Table 1, albeit with very minor increase in the error for 1 and 2 fiber configurations.

**Table 2.** Performance of the classifier for standard model.

Ground truth (Standard Model)	1 Fiber	2 Fibers	3 Fibers
Misclassification (SNR = 3) (Our Approach)	4.2%	4.8%	5.1%

**Fig. 3.**
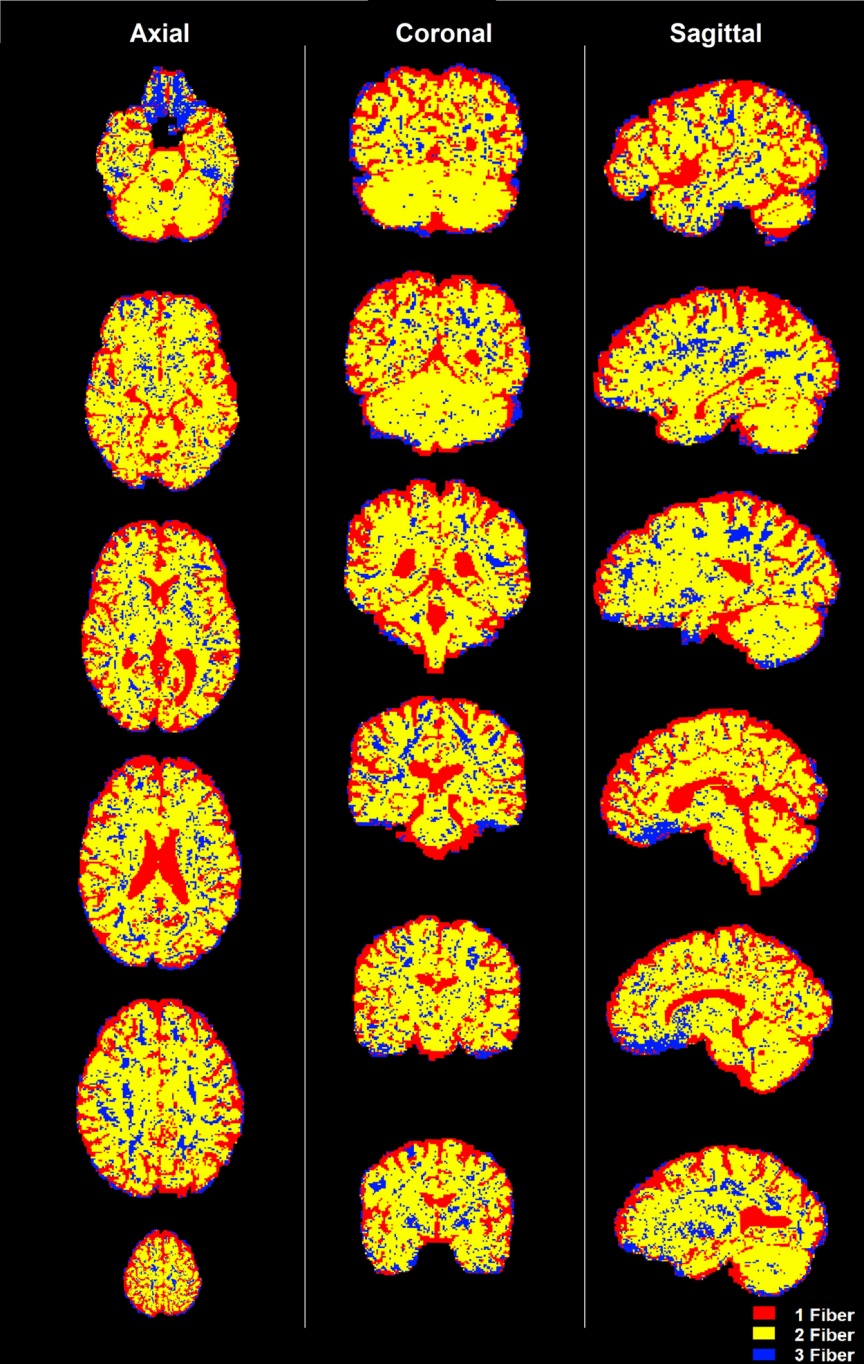
Classification of voxels in three classes (1, 2, or 3 fibers) in in-vivo data from the HCP dataset. Each slice is 10 voxels away from the surrounding slices in a specific column.

We also tested our algorithm on in-vivo human data from the Human Connectome Project (HCP) (Van Essen et al., 2013) as shown in Figure 3. As expected, most of the white matter had two fibers, with certain regions in the centrum semiovale having 3-fiber crossings, which is known from anatomical validation studies. Also note that, the cerebrospinal fluid (CSF) and ventricles show the existence of only one “fiber” population. We also note that, the central portion of the corpus callosum shows multiple fibers using our method. This is because we do not model fiber dispersion explicitly but instead rely on very small crossing angles (10 deg) to implicitly model fiber dispersion.

### 3.2 The posterior estimator performance

**Fig. 4.**
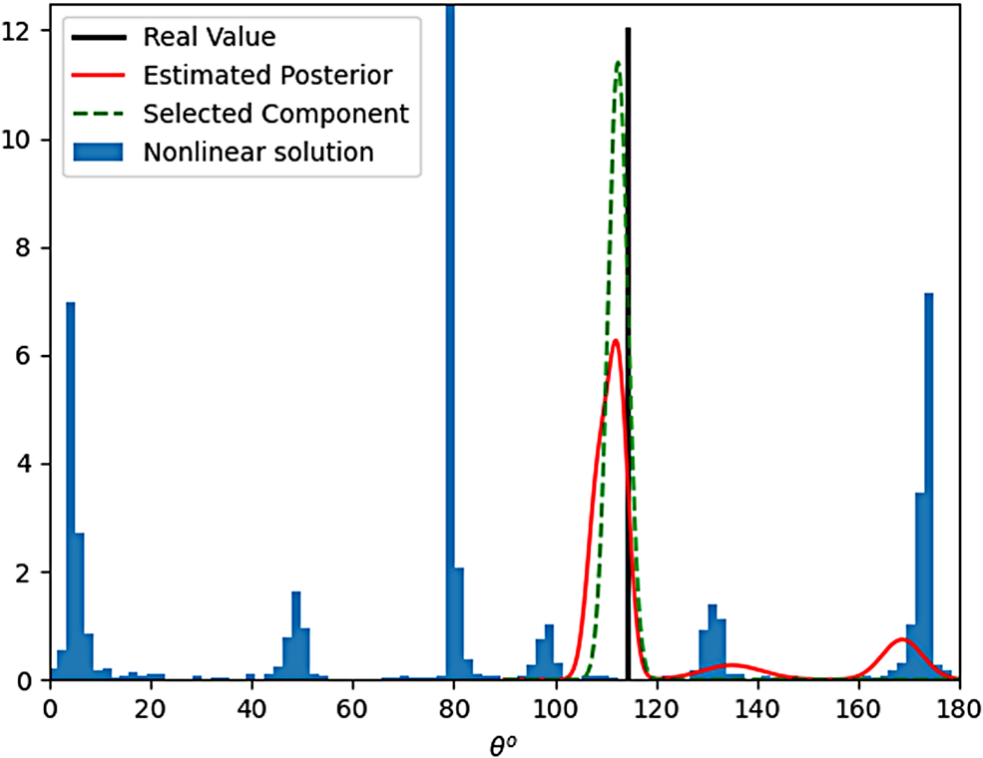
Estimation of θ. Black line indicate: true value, red curve: estimated posterior, green curve: selected component, blue histogram: possible solution given by a nonlinear estimator.

We train three different posterior networks based on the underlying number of fibers (one, two or three). In this subsection, we evaluate the performance of our posterior estimator on 20,000 test samples (noisy with SNR = 3). The output of each network will be $\psi =\left\{w_{i},\mu_{i},\Sigma_{i}\right\}$. Figure 4 demonstrates the estimated posterior (red curve) for the fiber orientation corresponding to angle $\theta_{1}$. The true value ($\theta_{1}=116^{o}$) is marked with a black line. As can be seen, the posterior has multiple modes indicating several plausible solutions. Due to the multi-modal nature of the posterior distribution, naive calculation of the mean of the distribution would give erroneous results. Further, in addition to the posterior estimation, we also need to find the final (mean) estimate of the model parameters (the fiber orientation in this case). Therefore, we choose the dominant Gaussian component (green curve) of the posterior as our “true component”. To select the most appropriate component, we first calculate $\delta_{i}=\frac{w_{i}}{trace(\Sigma_{i}^{0.5})}$. Then, the component corresponding to the largest $\delta_{i}$ represents our final estimate or the most dominant component to use. This method to calculate the dominant component ensures that a component with very large variance representing a fit to the noise in the data is discarded. For the particular case at hand, we see that the peak of the dominant component is very close to the ground truth. Further note that, the variance of this parameter also provides the uncertainty in the estimation of this parameter. This unique information is not provided by any other estimation technique (unless one runs a monte-carlo simulation by initializing a non-linear estimator with a large number of starting points to obtain all possible values of the estimated parameter). On the other hand, the blue histogram in Figure 4 corresponds to the possible outcomes of the Levenberg–Marquardt (LM) nonlinear estimation algorithm that provides a solution to equation (1) for different starting points. In other words, every time we run the nonlinear approach, a different value (based on the frequency given by the histogram) in the region marked by the histogram will be given as the estimate of θ. This histogram plot implies a lack of robustness and repeatability due to noise in the data.

Alternatively, one could use a brute force approach and use large number of initializations to arrive at a solution that gives the lowest error. However, this method is fraught with issues when the solution is highly multi-modal in nature, as explored in Jelescu et al. (2016). Further, the amount of computations required (up to several weeks for an HCP style acquisition) using such an approach would make it almost impossible to use it in more practical scenarios covering the entire brain. Additionally, we would like to point to the work in Sjölund et al. (2018), where the authors study the effect of noise on estimation of dMRI-derived parameters. We also note that the nonlinear approaches only provide a point estimate, and not uncertainty quantification.

**Fig. 5.**
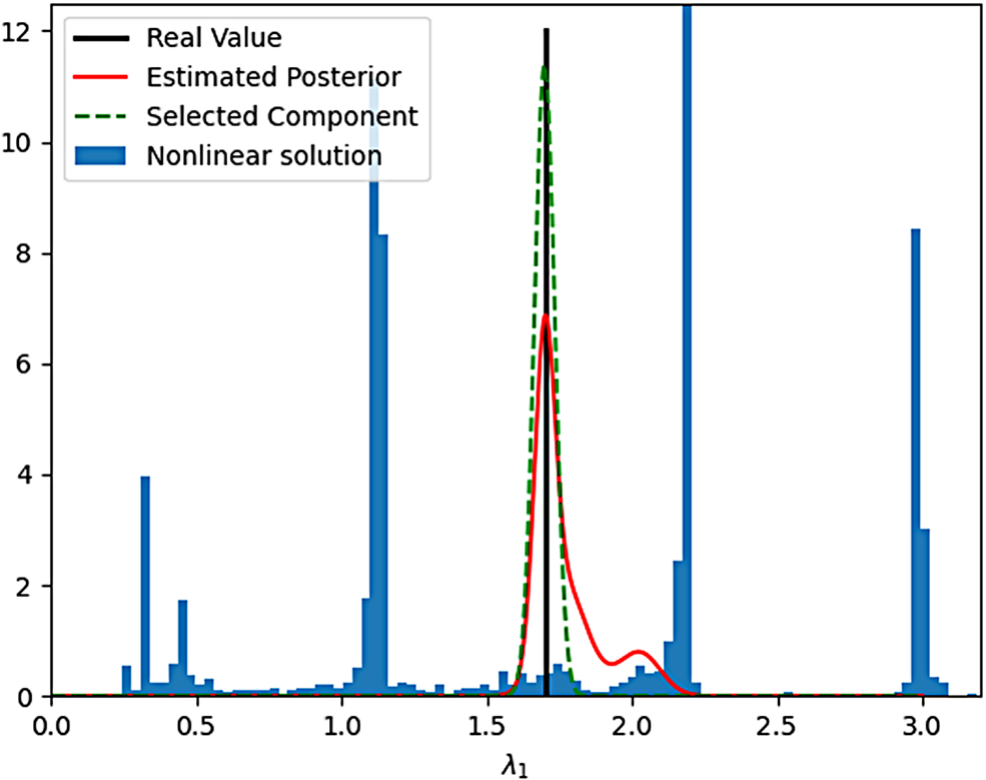
Posterior estimation of the eigenvalue $\lambda_{11}$. Black line indicates true value, red curve: estimated full posterior, green curve: selected component, blue histogram: possible solution range given by a nonlinear estimator.

As another example, Figure 5 demonstrates the estimated posterior for the eigenvalue $\lambda_{11}$. In this example, the voxel contains two fibers $x=[\theta_{1}=\pi /6,\;\;\phi_{1}=\pi /3,\;\;\lambda_{11}=$$1.9,\;\;\;\;\lambda_{21}=1.35,\;\;\lambda_{31}=0.5,\;\;\lambda_{41}=0.25,\;\;\theta_{2}=pi/5,\;\;\phi_{2}=7\pi /4,\;\;\lambda_{21}$$=1.7,\;\;\lambda_{22}=1.06,\;\;\lambda_{32}=0.5,\;\;\lambda_{42}=0.25\;]$. In Figure 5, the red curve is the full posterior while the green curve represents the dominant component in the posterior. It is clear that the true value is very close to the mode of the selected component, while the possible values given by the LM method (histogram) give several solutions throughout the range of $\lambda_{1}$.

**Fig. 6.**
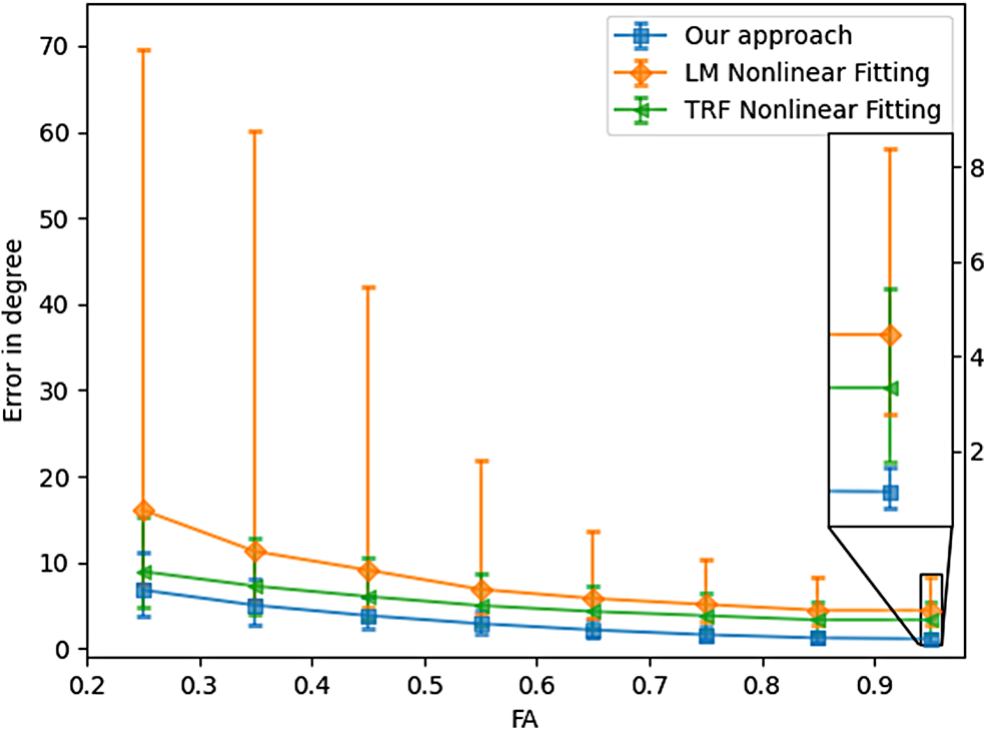
Angular error as a function of minimum FA (of the fast component) among the multiple fibers.

**Fig. 7.**
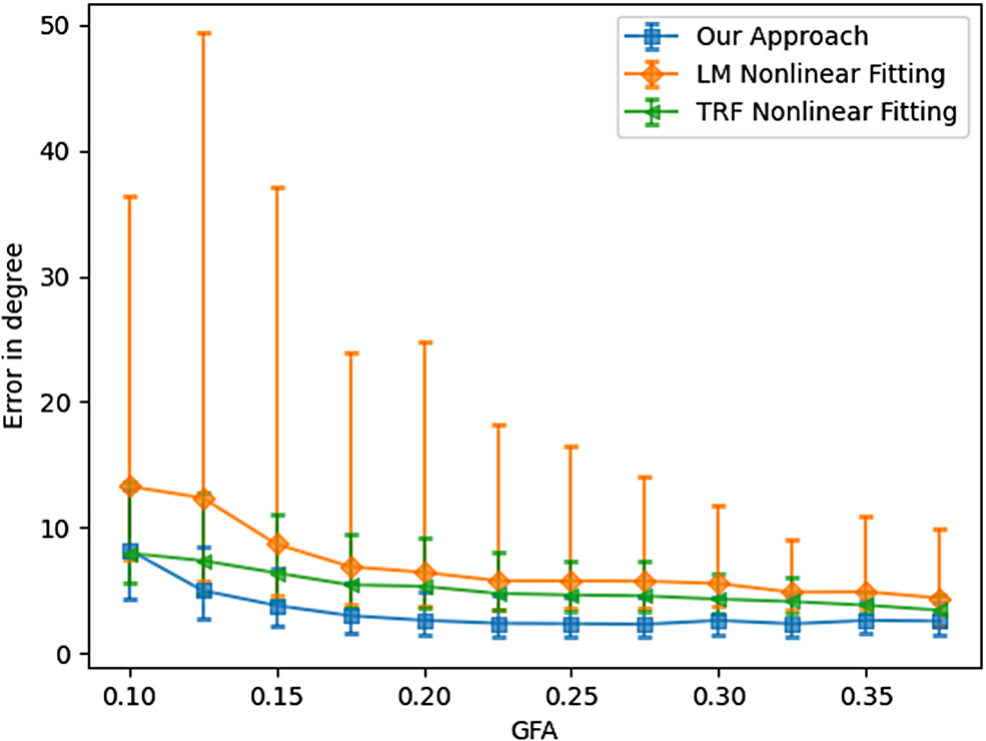
Angular error as a function of GFA of the signal at b = $1000\;\;s\;/\;mm^{2}$.

**Fig. 8.**
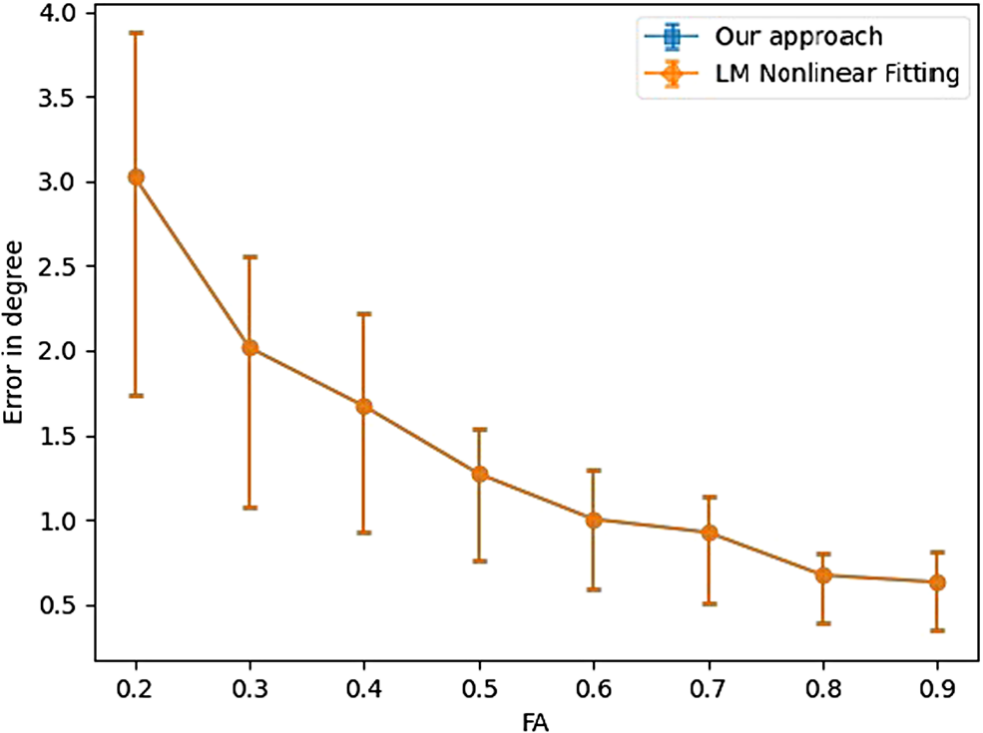
Angular error as a function of FA for the case of a single fiber.

To evaluate the accuracy across all the 20,000 samples, Figures 6 and 7 depict the angular errors as a function of the ground truth FA and GFA respectively. In this plot, the error bars indicate 25 and 75 percentile, and the midpoint is the median of the errors. We compare our algorithm with two nonlinear approaches: 1) Levenberg-Marquardt (LM) and 2) Trust Region Reflective (TRF) (with bounds on the range of values the parameters can take). An important point to note for these nonlinear methods is that we fix the number of fibers *a-priori* (and hence the number of parameters to estimate is known) assuming this information is accurately known from other techniques (e.g., CSD or our classifier approach presented earlier). For all the FA values, our method outperforms both the LM and TRF nonlinear estimators in terms of the median. Note however, that both the non-linear estimators have a much larger variance indicating a large number of outlier estimations using these methods. On the other hand, the proposed method has very small error bars (and low median error) for all the simulated samples. As expected, the error has an inverse relationship with FA, that is, all methods perform better if the tensor becomes more anisotropic. It should be noted that the fiber orientation is estimated more precisely by all methods when the underlying voxel contains only one fiber configuration (see Fig. 8).

**Fig. 9.**
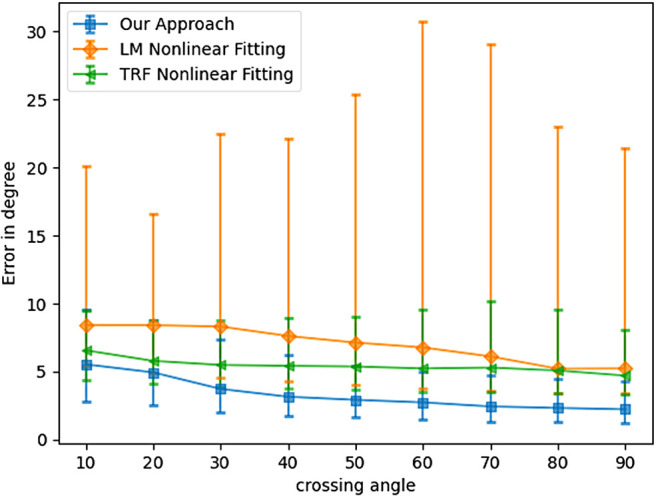
Angular error as a function of the crossing angle between fibers.

For the multi-fiber voxels, the angle between the fibers (known as the crossing angle) may affect reconstruction accuracy. For example, Malcolm et al. (2010) show that some classical techniques (e.g., sharpened spherical harmonics) fail to resolve two fiber crossings when the crossing angle is below a certain value (e.g., 30 degrees). To show the robustness of our method in recovering small crossing angles, we demonstrate the angular error as a function of the crossing angle between two fibers in Figure 9. As can be seen, the proposed method accurately recovers fiber orientations even at small crossing angles (less than 30 degrees). This is also a significant advantage of our method, which can be very useful for tractography algorithms. An important factor to note here is that for the nonlinear methods, we fixed the number of estimated parameters as the number of fibers in the signal was assumed to be known. However, as shown earlier in this work (Table 1), standard techniques like CSD often fail to estimate the accurate number of fiber crossings at such low angles, which can significantly affect the results of the nonlinear techniques. Thus, the results for nonlinear techniques are true only if the estimate of number of fibers is correct in all test samples, which is unrealistic using existing methods.

**Fig. 10.**
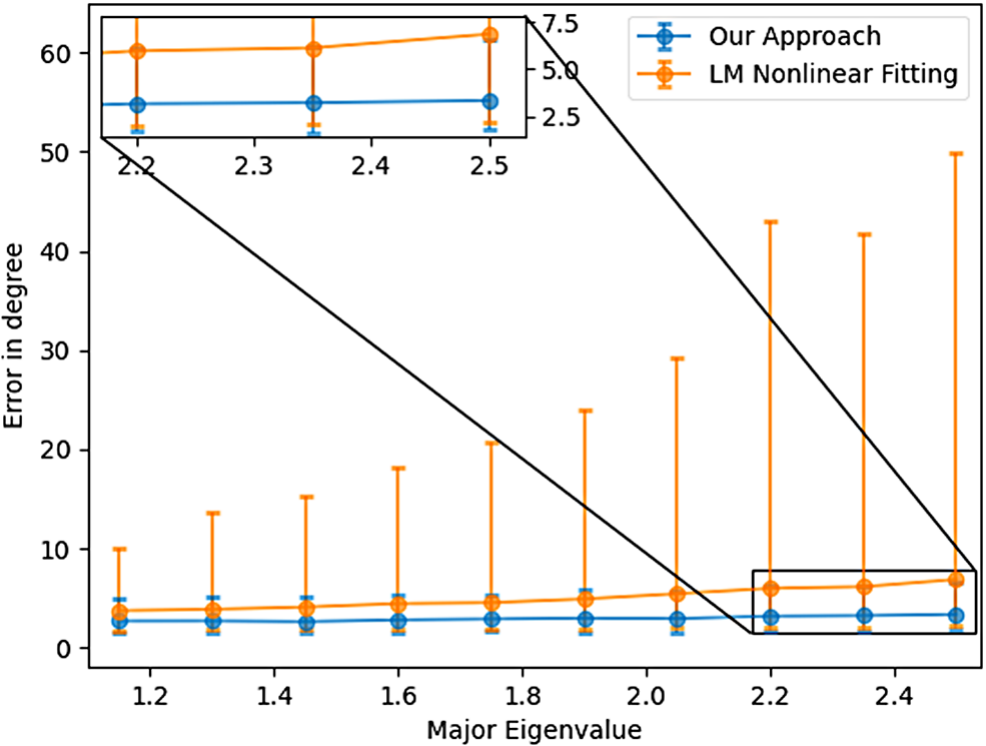
Angular error as a function of the largest eigenvalue.

**Fig. 11.**
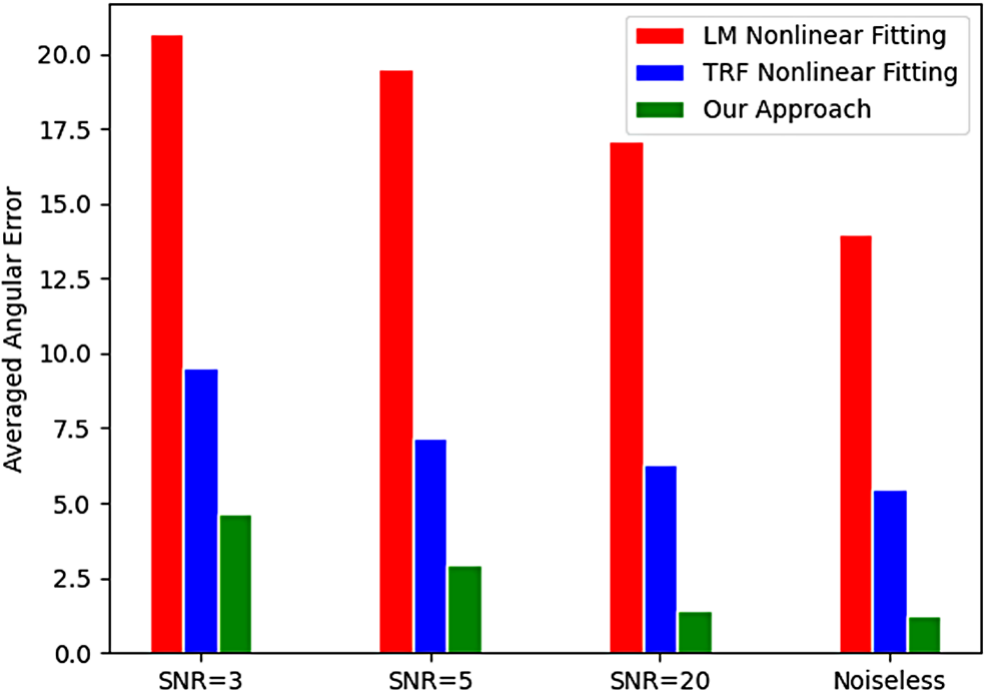
Average angular error for different measurement noise levels.

**Fig. 12.**
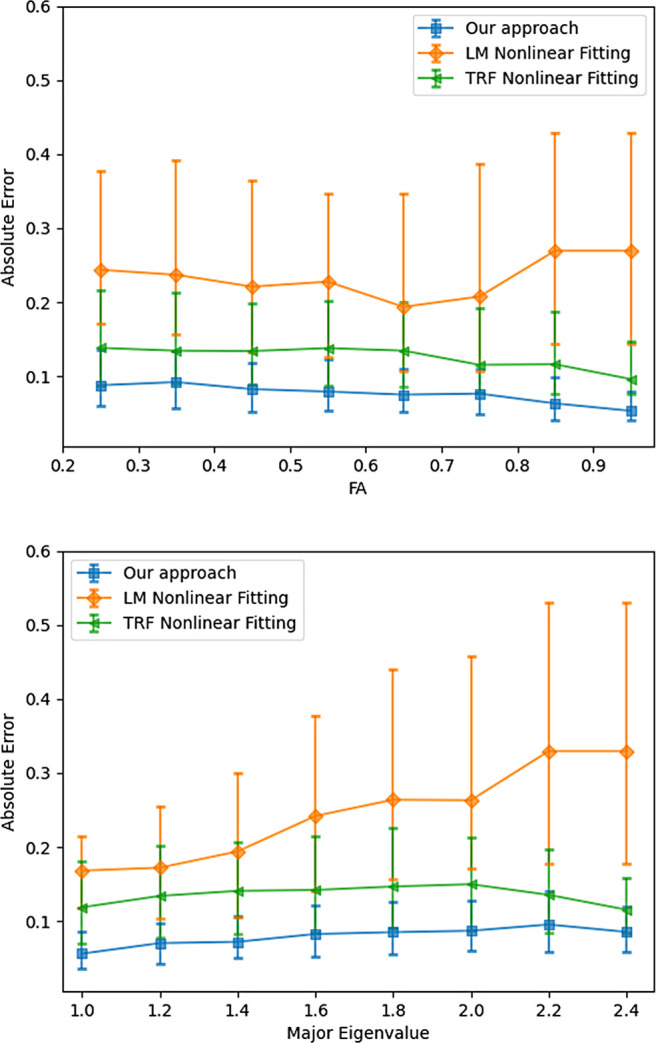
Error in estimation of the largest eigenvalue as a function of the ground truth FA of the fast component (top) and the largest (major) eigenvalue (bottom).

**Fig. 13.**
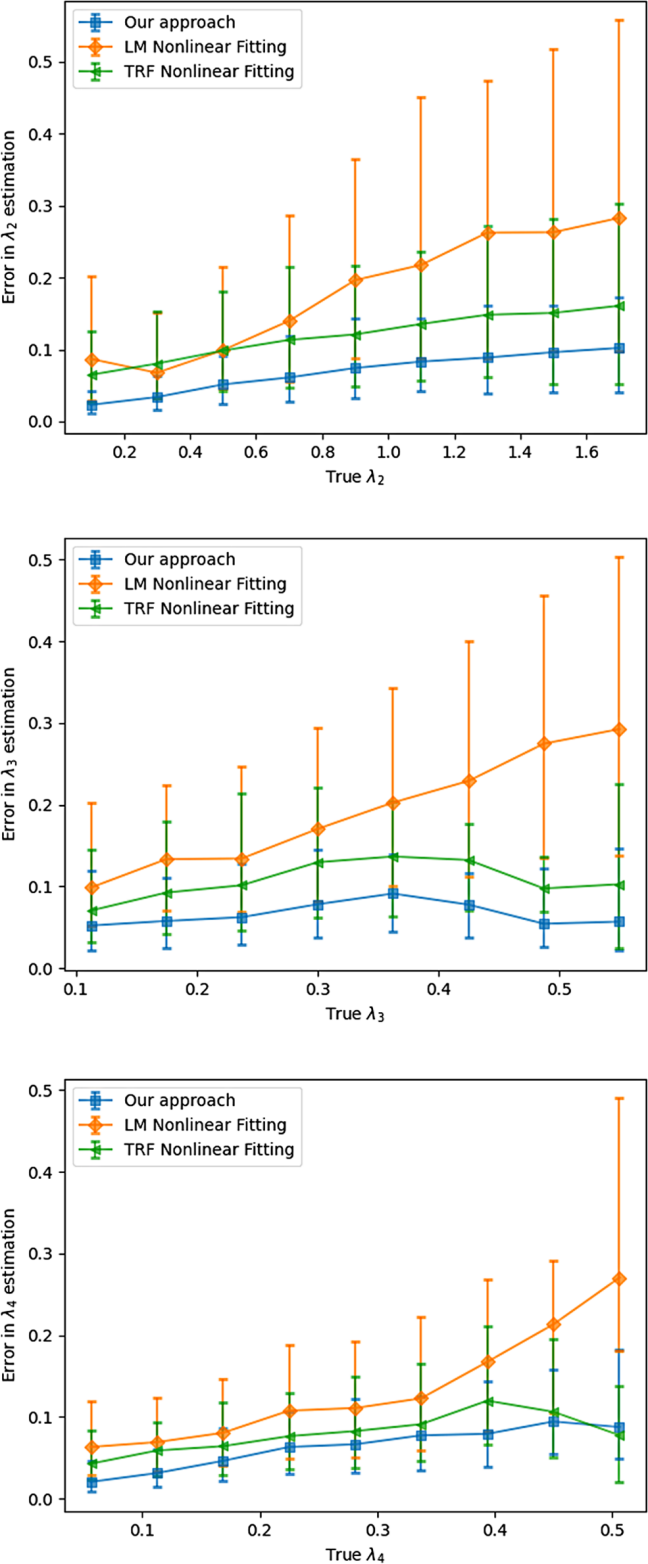
Error in estimation of the eigenvalues $\lambda_{2},\;\;\lambda_{3}$ and $\lambda_{4}$.

**Fig. 14.**
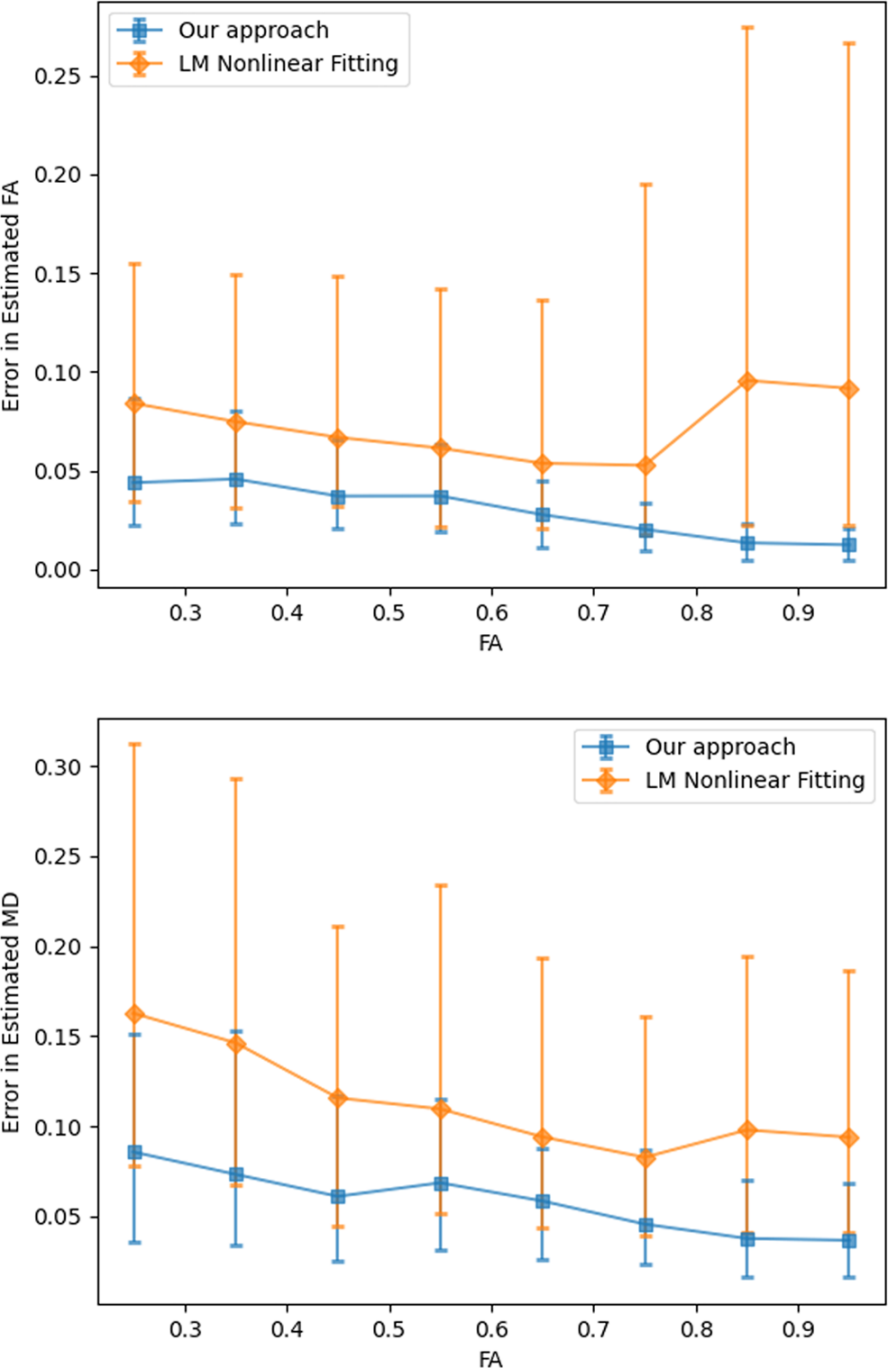
Top: Error in estimated fractional anisotropy compared to ground truth FA. Bottom: Error in estimated mean diffusivity as a function of ground truth FA.

Figure 10 shows the relationship between angular error and the magnitude of the largest eigenvalue. While the proposed method is robust to changes in the eigenvalue, the nonlinear estimator fails in many more cases leading to larger number of voxels being estimated incorrectly. Figure 11 show average angular errors for different levels of measurement noise. The figure confirms the efficacy of our approach for different noise levels. In Figure 12, we show the average absolute error in the estimation of the largest eigenvalue as a function of the ground-truth FA of the fast component (top), and the largest eigenvalue $\lambda_{1}$ (bottom). Clearly, our method outperforms the other two nonlinear techniques by a large margin. Similarly, Figure 13 shows the error in the other three eigenvalues. Figure 14 shows the error in the estimated FA and mean diffusivity (MD) respectively as a function of FA. In all the cases, our approach shows lower error compared to the nonlinear fitting method and results in more robust results in the presence of high noise.

**Fig. 15.**
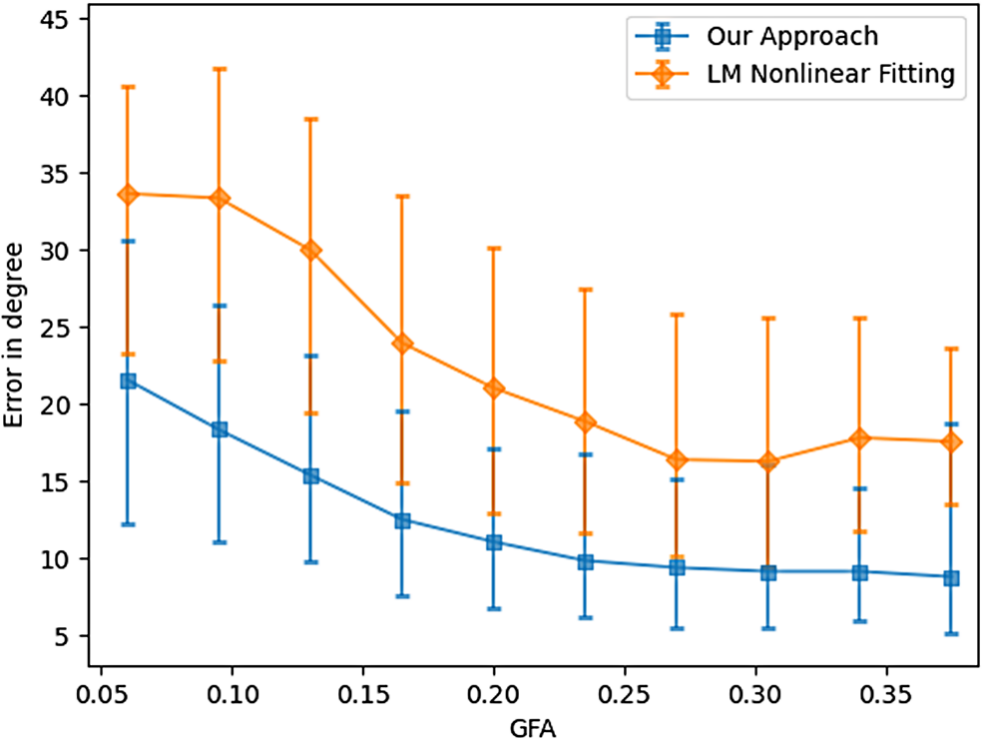
Angular error for three fiber cases with respect to GFA.

Estimation of three fiber crossings is always challenging. Figure 15 validates the efficacy of our approach of posterior estimation in a voxel containing three fiber crossings. The x-axis is the GFA of the voxel for the three fiber configuration. Although we assumed a fixed value for the weights of the fast and slow compartments at the training stage, the weights may vary in reality. To capture the effect of this difference in the model, Table 3 shows the angular error when the underlying weights of the fast and slow components are different from the weights we used in the simulated training dataset (0.7 and 0.3 respectively). Nevertheless, our method shows excellent robustness and generalization ability to such out-of-distribution test samples. As can be seen, while this difference does not affect the angular error, we see slightly higher error in the estimation of the eigenvalues (total error calculated as the sum of the errors in estimation of all the eigenvalues) which is still comparable to the errors obtained using other nonlinear methods.

**Table 3.** Performance with different model parameters.

Weights (fast - slow)	Angular error (degree)	Absolute total Eigenvalue error ($\mu m^{2}\;\;\;/\;ms$)
0.5-0.5	3.96	0.22
0.6-0.4	3.31	0.14
0.7-0.3	2.93	0.07
0.8-0.2	3.15	0.12
0.9-0.1	3.91	0.19

We would like to note that the machine-learning algorithm was trained on the entire feasible range of values for each of the model parameters. For example, the range of values for the fast diffusivities ranges from $\left[1,3\right]\;\mu m^{2}\;/\;ms$ whereas the slow diffusivities range from $\left[0.1,0.6\right]\;\mu m^{2}\;/\;ms$. Similarly, the orientations are spread across the sphere while the volume fractions for each fiber varies from 0.1 to 0.9. While these values are within the possible set of values observed in human data even in pathological cases, the test data set was not limited to this range. Yet, as shown in Figures 7, 8, 10, and 11, our method was able to provide low error rates for these settings. To further demonstrate our work’s generalization ability, we also used the proposed framework with the standard model of diffusion (instead of the biexponential model), details of which can be found in H.S. Karimi et al. (2023).

**Fig. 16.**
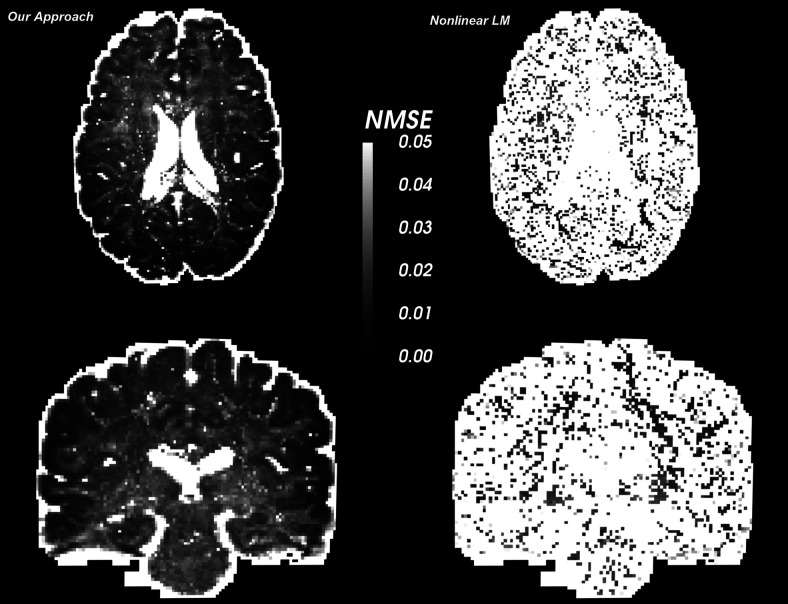
NMSE in signal reconstruction in a subject from HCP database. Left: our approach, Right: LM nonlinear fitting. Clearly, our method shows low fitting error (less than 5%).

Figure 16 shows the normalized mean squared error (NMSE) in two slices in a human in-vivo dataset from the HCP database. We used all the 257 gradient directions at b-values of $\left\{1000,2000,3000\right\}\;\;s\;/\;mm^{2}$ and estimated the model parameters voxel-wise across the whole brain. The NMSE was then calculated at each voxel using the estimated parameters to generate the signal using the forward model. A similar approach was used for the LM method (right column) as well, with the number of fibers assumed to be correctly known from our classifier. Note that, this model selection problem is already known to be error prone; however, we used the voxel classifier developed in this work for the nonlinear method as well to show comparative results. For the proposed method, the NMSE is well below 0.05 (or less than 5% error in fitting) whereas it is much higher for the LM nonlinear estimator.

**Fig. 17.**
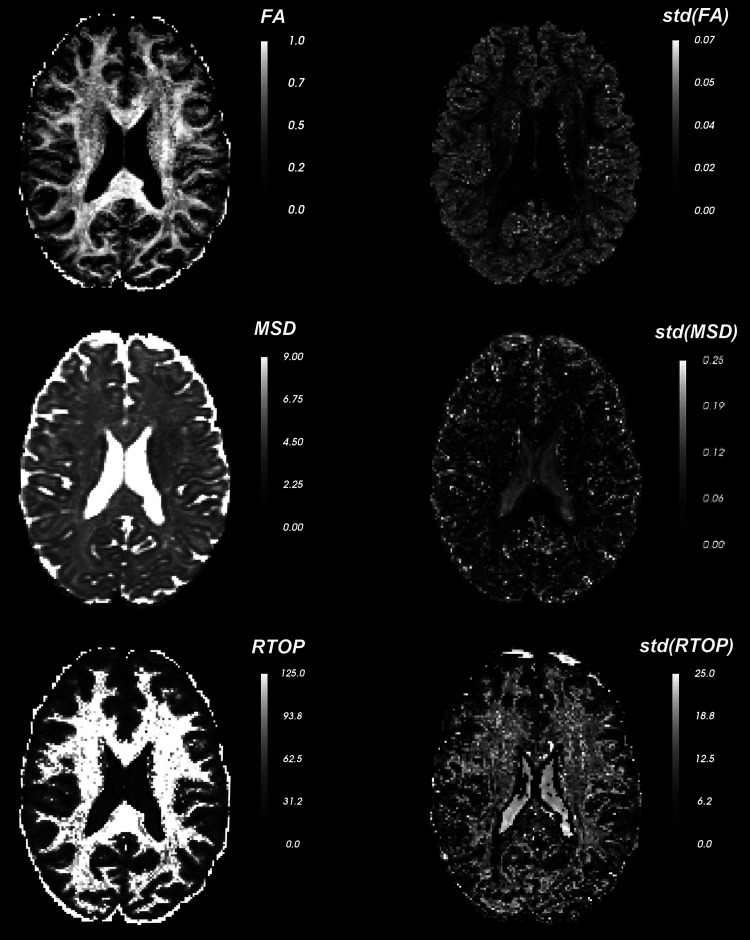
Axial slice shows the dMRI-derived measures (FA, MSD, RTOP) and the corresponding voxel-wise uncertainty in these measures quantified using standard deviation (std).

**Fig. 18.**
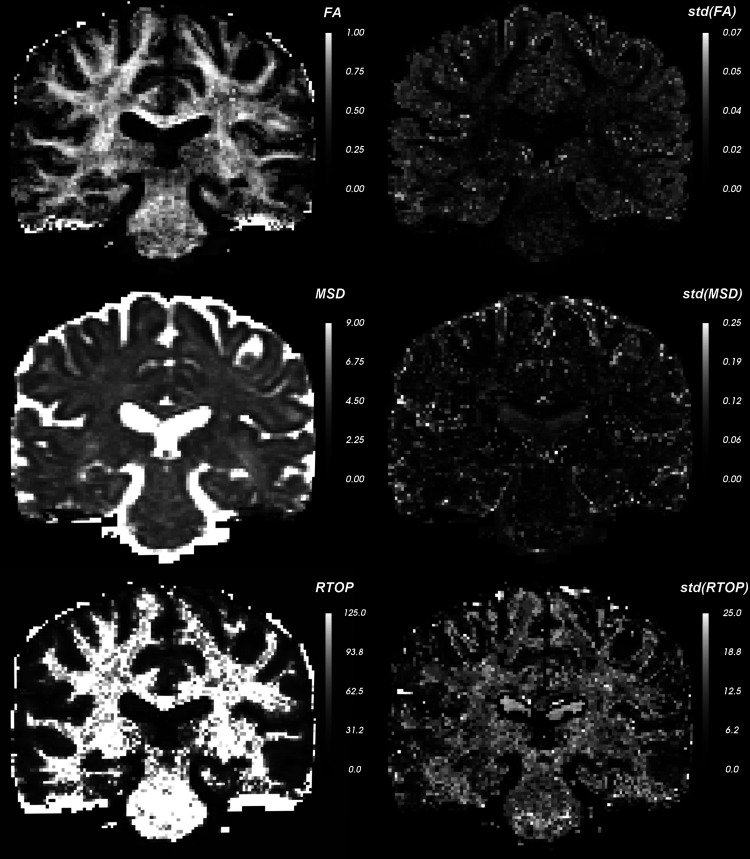
Coronal slice shows the dMRI-derived measures (FA, MSD, RTOP) and the corresponding voxel-wise uncertainty in these measures quantified using standard deviation (std).

**Fig. 19.**
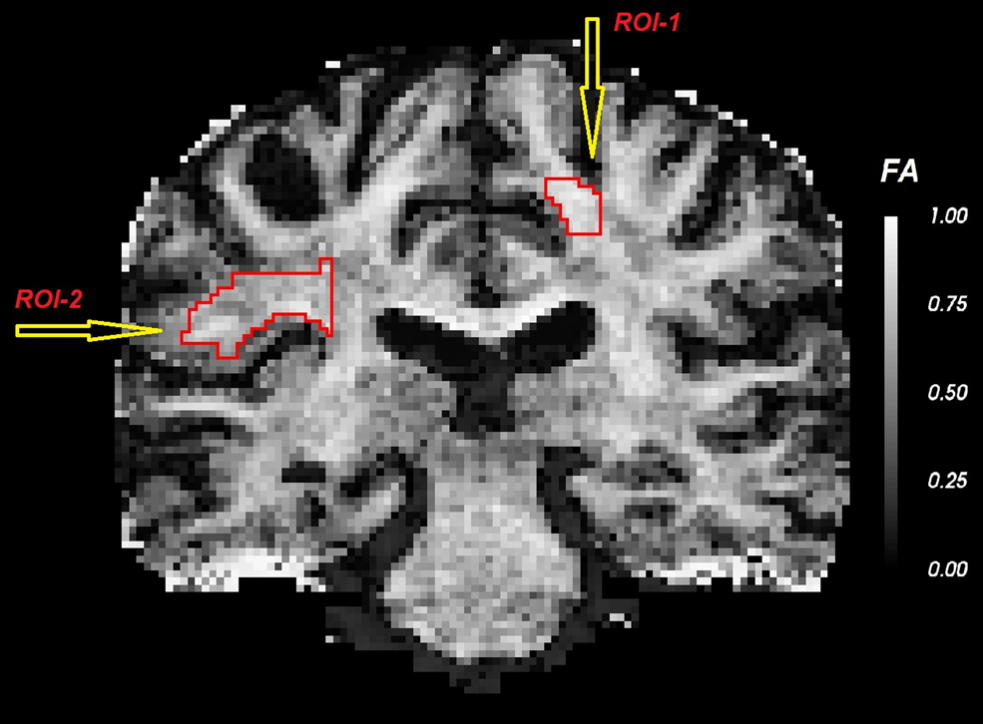
Two regions of interest (ROIs) for comparing arithmetic mean and variance-weighted mean.

In Figures 17 and 18, we compute three dMRI measures, namely FA (of the fast diffusing component), MSD, and RTOP as well as their uncertainty in estimation (quantified using the standard deviation). We estimate the uncertainty in the derived measures from the uncertainty in the estimation of the eigenvalues using the unscented transform (Julier & Uhlmann, 1997) (see derivation details in Appendix). We see that the FA estimate is highly uncertain in the gray matter regions, whereas MSD is more uncertain in the CSF areas. Also note that, several boundary voxels (ventricles and surrounding tissue) show high uncertainty (variance) in all the measures indicating that those values cannot be trusted in statistical analyses involving group studies. To demonstrate how uncertainty can be used in neuroimaging studies, we first choose a region of interest (ROI) in the brain. Figure 19 shows two distinct ROIs enclosed by a red curve. Next, we calculate the mean of the dMRI measures via two methods: 1) standard arithmetic average where all data equally contribute to the final mean, and 2) variance-weighted average where the weights of each data point in calculating the final mean are computed by the inverse of their variance. We summarize these average values in Table 4 for FA, MSD, and RTOP. Clearly, there is a considerable difference between these values which highlights the importance of uncertainty quantification.

**Table 4.** Variance-weighted average vs. arithmetic average.

dMRI Measure	FA	MSD	RTOP
Arithmetic Average ROI-1	0.829	2.97	329
Variance-weighted Average ROI-1	0.886	3.01	263
Arithmetic Average ROI-2	0.702	3.06	435
Variance-weighted Average ROI-2	0.916	3.01	262

One of the other motivations of this work is to find a parameter estimation technique that is more time-efficient. Table 5 indicates the required computational times for executing the introduced methods for the whole brain (HCP dataset at $1.25^{3}\;\;mm^{2}$ spatial resolution). The results confirm a huge improvement (a factor of 400-fold reduction in computation time) using our method that avoids the long computational time requirements for the nonlinear techniques. For all the methods in Table 5, we use 20 CPU (2.2 GHz) cores. Training of the NN was however done on machine with 2 GPU cores with 22 GB memory.

**Table 5.** The required time.

Approach	Nonlinear LM	Nonlinear TRF	Our Method
Consumed Time	967 hours	1105 hours	2.41 hours

## 4 Conclusion

Accurate parameter estimation and uncertainty quantification in dMRI models is necessary for robust statistical analyses in neuroimaging studies. In this paper, for the first time, we estimate the full posterior distribution of the underlying parameters without any likelihood computation. Unlike most existing methods that give only point estimates, our approach can estimate the uncertainty of parameters and the derived measures. Furthermore, we developed a DNN technique to accurately classify the number of fibers in the brain. In terms of point estimation, our method is more robust against noise compared to the nonlinear approaches. Additionally, the proposed approach is extremely fast compared to standard nonlinear techniques, making it ideal for various large-scale applications.

## Data Availability

The imaging data used in work are available from the Human Connectome Project (HCP) website (Van Essen et al., 2013). The codes for the presented techniques are available on the following repository: https://github.com/hazhars/posterior_dMRI.

## Appendix

Originally proposed by Julier and Uhlmann (1997), the unscented transform aims at finding the first- and second-order statistics of a function undergoing a nonlinear transformation. In this paper, we use this transform to estimate the statistics of dMRI measures, which are nonlinear functions (denoted by f(⋅)) of eigenvalues. The estimated posterior distribution of the eigenvalues is a Gaussian distribution $\lambda \sim N\left(\mu_{\lambda },\;\Sigma_{\lambda }\right)$. Our goal is to find the mean and covariance of the derived measures $\left(\mu_{M},\;\Sigma_{M}\right)$ using the unscented transform. To this end, we first choose a set of sigma points $\chi_{k},k\in \left\{0,...,2D+1\right\}$, where D is the dimension of λ. To compute the sigma points, we first find the scaled Cholesky decomposition L of the parameters covariance matrix, $L=cholesky\left(D\Sigma_{\lambda }\right)$. Subsequently, the sigma points, and their associated convex weights are,

$$\chi_{0}=\;\mu_{\lambda }$$

$$\chi_{j}=\mu_{\lambda }+\;\;L_{:j},\;\;j\in \left\{0,...,D\right\}$$

$$\chi_{j+D}=\mu_{\lambda }-L_{:j},\;\;j\in \left\{0,...,D\right\}$$
(9)

$$w_{0}=\frac{\kappa }{D+\kappa }$$

$$w_{j}=\frac{1}{2\left(D+\kappa \right)},\;\;j\in \left\{1,...,2D+1\right\}$$
(10)

where $L_{:j}$ is the $j^{th}$ column of matrix L. In (10), κ serves as an adjustable scaling parameter. Then, we calculate the nonlinear transform of each sigma point:

$$M_{k}=f\left(\chi_{k}\right),$$
(11)

where f(⋅) represents any of the derived measures given in equations (7) or (8). Finally, the transformed distribution can be calculated using the following equations:

$$\mu_{M}\sum_{k}w_{k}M_{k}$$
(12)

$$\sum_{M}=\sum_{k}w_{k}(M_{k}-\mu_{M}){\left(M_{k}-\mu_{M}\right)}^{T}.$$
(13)

**Remark.** *The sigma points for the eigenvalues may contain some negative values especially if the elements in λ are close to zero. However, since a negative eigenvalue is meaningless in our case, we enforce a constraint to ensure that all the sigma points fall in the positive region. We apply this constraint by projecting the unconstrained sigma points into positive values using the following quadratic programming problem:*

$$\underset{{\hat{\chi }}_{k}}{min}{\left(\chi_{k}-{\hat{\chi }}_{k}\right)}^{T}\Sigma_{\lambda }^{-1}\left(\chi_{k}-{\hat{\chi }}_{k}\right)$$
(14)

$$subjectto:-{\hat{\chi }}_{k}<0.$$

*Afterwards, ${\hat{\chi }}_{k}$ serve as new sigma points that do not contain any negative value.*
